# Transformation of a Cutaneous Follicle Center Lymphoma to a Diffuse Large B-Cell Lymphoma—An Unusual Presentation

**DOI:** 10.1155/2010/296523

**Published:** 2010-05-31

**Authors:** J. Dias Coelho, F. Diamantino, I. Costa, P. Farinha, P. Gameiro, M. Sebastião, J. Baptista

**Affiliations:** ^1^Department of Dermatology, Centro Hospitalar Lisboa Central, EPE, Alameda Santo António dos Capuchos, 1169-050 Lisbon, Portugal; ^2^Department of Hematology, Centro Hospitalar Lisboa Central, EPE, Alameda Santo António dos Capuchos, Lisbon, Portugal; ^3^Department of Pathology, Centro Hospitalar Lisboa Central, EPE, Rua José António Serrano, 1150-199 Lisbon, Portugal; ^4^Laboratory of Hemato-Oncology, Instituto Português de Oncologia de Lisboa Francisco Gentil, EPE, Rua Professor Lima Basto, 1099-023 Lisbon, Portugal

## Abstract

Primary cutaneous follicle center lymphoma (PCFCL) is characterized by a proliferation of follicle center cells in the skin. A definitive diagnosis is frequently delayed because of difficulties in interpretation of the histopathologic findings. It has an excellent prognosis with a 5-year survival over 95% and its risk of transformation has not been established. We describe a case report of man with a gastric diffuse large B-cell lymphoma (DLBCL) referred to our clinic because of nodules in the back that had gradually developed over a period of 10 years. A biopsy performed 3 years before was interpreted as reactive follicular hyperplasia. A new skin biopsy revealed a diffuse large B-cell lymphoma and immunoglobulin heavy chain gene rearrangements from the initial skin biopsy (PCBCL) and the DLBCL gastric biopsy were studied by polymerase chain reaction and an identical clonal rearrangement was detected which was highly suggestive of a transformation lymphoma.

## 1. Case Report

Primary cutaneous follicle center lymphoma (PCFCL) is characterized by a proliferation of follicle center cells (centrocytes and centroblasts) with a follicular, follicular and diffuse, or diffuse growth pattern [1]. It shows a predilection for the scalp, forehead, and trunk and dissemination to extracutaneous sites rarely occurs [2]. Transformation of systemic follicular lymphoma (FL) into aggressive non-Hodgkin^,^s Lymphoma is associated with poor prognosis and has been reported with a wide range of frequency (range between 10%–70%). While the annual risk of transformation of systemic FL is 3%, PCFCL has an excellent prognosis with a 5-year survival over 95% [1] and its risk of transformation has not been established [3].

**Figure 1:**
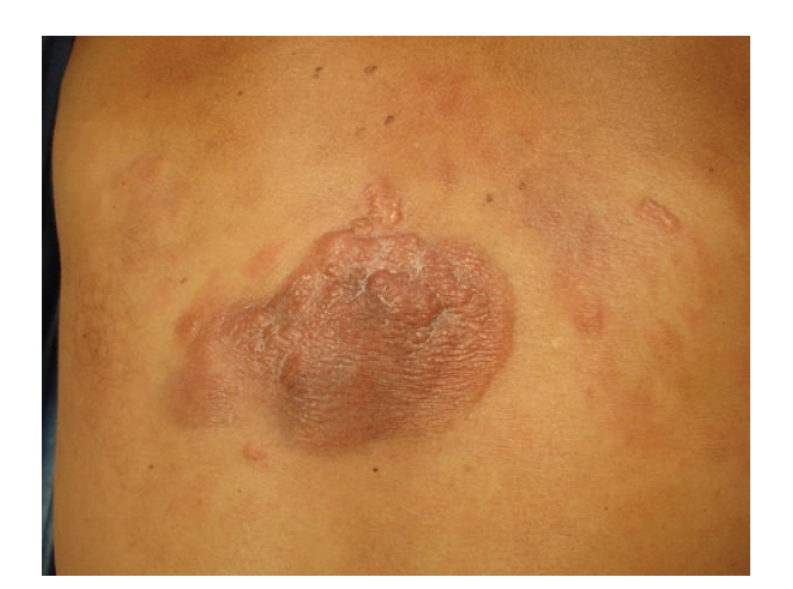
Multiple erythematous to purple, sharply demarcated pruritic nodules in the back that enlarged and coalesced in the last year originating a tumor measuring 11 × 7 cm in diameter with some satellite lesions.

We describe a case report of a 44-year-old white man with a gastric diffuse large B-cell lymphoma (DLBCL) referred to our clinic by the hematology department because of multiple erythematous to purple, sharply demarcated pruritic nodules in the back that enlarged and coalesced in the previous year originating a tumor measuring 11 × 7 cm in diameter (Figure 1). These lesions had gradually developed over a period of 10 years and a biopsy of one of the nodules was diagnosed in a different hospital as reactive follicular hyperplasia. These findings were interpreted as an inflammatory pseudolymphomatous reaction. He received treatment with tetracyclines and had improvement after sun exposure.

**Figure 2:**
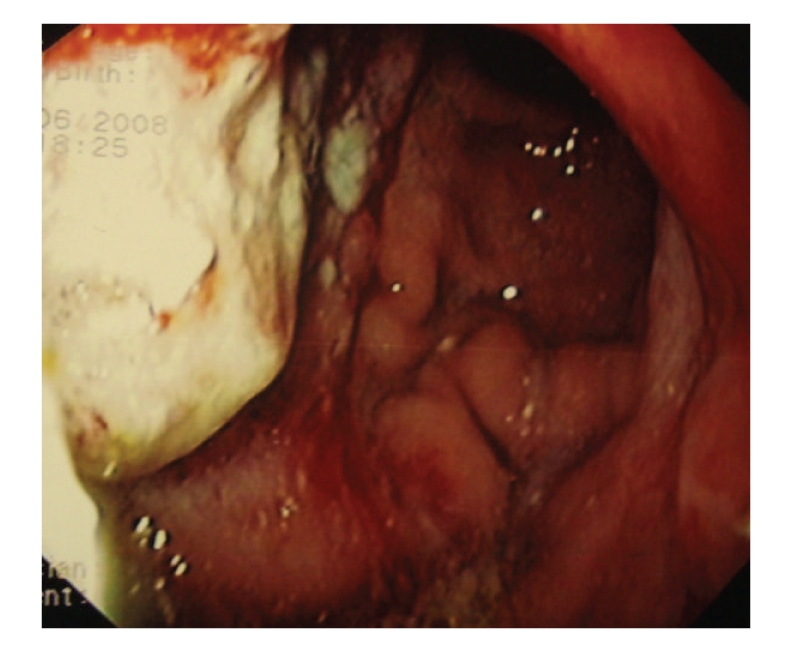
Endoscopy revealed two gastric ulcers. Biopsy revealed a DLBCL.

**Figure 3:**
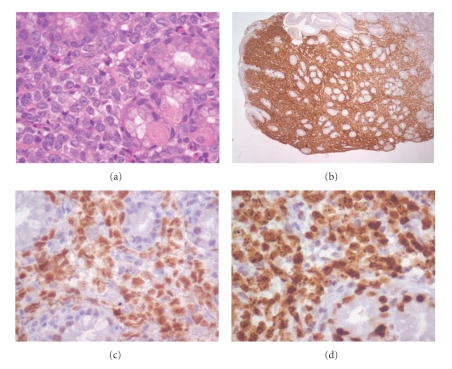
Stomach biopsy: (a) H&EX40 (Olympus BX40)—DLBCL, (b) antibody staining CD 20+, (c) antibody staining BCL6+, (d) antibody staining Ki67+.

**Figure 4:**
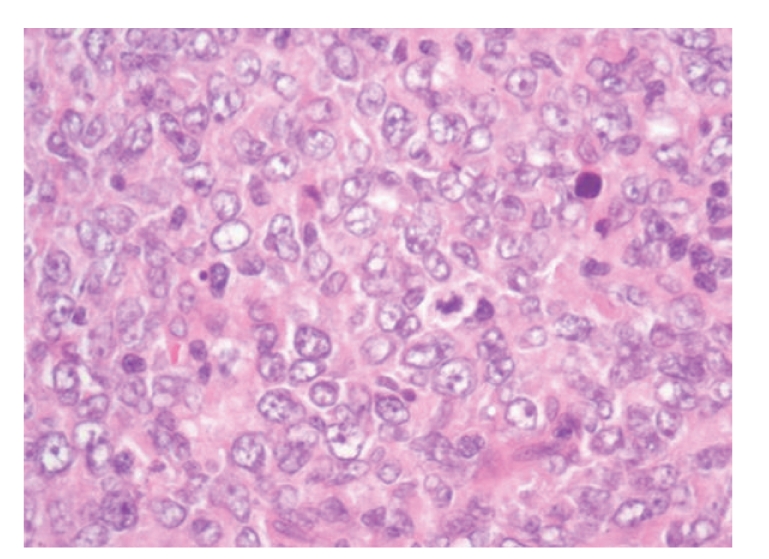
Skin biopsy 2009: H&EX40 (Olympus BX40) —DLBCL.

Recently, he was referred for an upper endoscopy because of epigastric pain and weight loss. Endoscopy revealed two gastric ulcers (H. Pylori positive) (Figure 2) and a computer tomography showed both mural thickening of the gastric mucosa and mediastinal and axilar lymphadenopathy. A gastric biopsy revealed a DLBCL (Figures 3(a)–3(d)). A bone marrow aspirate and biopsy showed minimal infiltration by low-grade B-cell lymphoma. A new biopsy of the skin lesions was performed and demonstrated diffuse infiltrate of confluent sheets of centroblasts, with many mitotic figures. The cells were CD20+, CD3−, CD30−, and BCL6+ and had a high proliferation rate (Ki67+), features compatible with DLBCL (Figure 4). The peripheral blood analysis revealed: normal beta-2-microglobulin (1,70 mg/L) and elevated LHD (571 U/L) levels. The lymphoma classification was DLBCL stage IV A; International Prognostic Index **(**IPI): 2.

**Figure 5:**
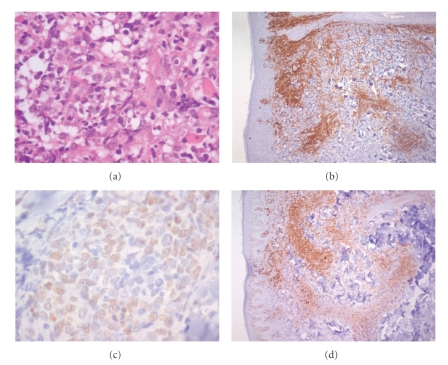
Skin biopsy 2006: (a) H&EX40 (Olympus BX40)—Primary cutaneous follicle center lymphoma, (b) antibody staining CD 20+, (c) antibody staining BCL6+, (d) antibody staining Ki67+.

**Figure 6:**
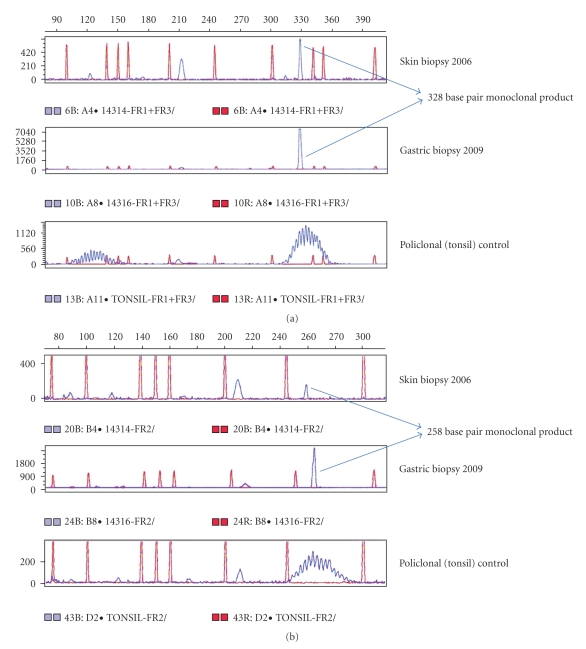
IgH clonality profiles from the PCFCL skin biopsy (2006) and the DLBCL gastric biopsy (2009). GeneScan results obtained using the BIOMED2: (a) FR1 and FR3 and (b) FR2 V_H_-J_H_ tubes (peaks in blue: patient and control DNA, peaks in red: TAMRA molecular weight marker). The same 328 base pair and 258 base pair monoclonal product is detected in both the skin and gastric biopsies, after FR1 (a) and FR2 (b) PCR amplification, respectively. No PCR product was detected after FR3 amplification (a) probably due to somatic hypermutation.

On re-examination, the skin biopsy obtained 3 years earlier was BCL2 weakly positive, CD10 negative, CD20 positive, and BCL6 positive and was reclassified as PCFCL (Figure 5). Immunoglobulin heavy chain gene (IgH) rearrangements were studied by polymerase chain reaction (PCR) using the Biomed2 strategy [4] in the earlier PCFCL and in the subsequent DLBCL biopsies of the skin and stomach, respectively. In each biopsy an identical clonal V_H_-J_H_ rearrangement was detected using FR1 and FR2 primers: 328 base pairs (bps) and 258 bps, respectively (Figures 6(a)–6(b)). 

The patient received six cycles of rituximab, cyclophosphamide, adriamycin, vincristine and prednisone (R-CHOP) chemotherapy, and omeprazole (20 mg once a day), with no improvement. Afterwards, he had undergone three different chemotherapy regimens (R-ESHAP, GEMROX, and IFM/VP16) with no response.

## 2. Discussion

PCFCL morphologic and phenotypic features can be similar or identical to those observed in secondary cutaneous involvement by systemic FL [5]. In our patient we cannot affirm with certainty that the initial lesion was a PCFCL since a complete staging investigation was not performed. However, the clinical history is very suggestive of Crosti^,^s lymphoma [5, 6]. In fact, as clearly shown in the WHO/EORTC classification of cutaneous lymphomas, PCFCL has many distinct features when compared with nodal FLs, as they are frequently negative for BCL2 and CD10 and only less than 25% of the cases have a BCL2 rearrangement. Thus, the most important diagnostic feature in both lesions is the BCL6+ (as skin marginal lymphomas are frequently BCL6 negative). Detection of an identical clonal V_H_-J_H_ rearrangement in both biopsies (skin and gastric) obtained at different time points and from different locations is compatible with the presence of the same B cell clone and strongly suggests transformation of the PCFCL into a DLBCL. 

PCFCL can infrequently progress into extracutaneous, but transformation into DLBCL has been rarely described [7]. 

The current case emphasizes that indolent cutaneous PCFCL can transform into high-grade lymphoma with poor outcome. An early diagnosis and proper treatment are essential in the management of PCFCL.
